# Respiratory evaluation through volumetric capnography among grade III obese and eutrophic individuals: a comparative study

**DOI:** 10.1590/1516-3180.2017.0085011017

**Published:** 2018-01-15

**Authors:** Débora Aparecida Oliveira Modena, Marcos Mello Moreira, Ilma Aparecida Paschoal, Mônica Corso Pereira, Luiz Cláudio Martins, Everton Cazzo, Elinton Adami Chaim

**Affiliations:** I PT, MSc. Doctoral Student, Universidade Estadual de Campinas (UNICAMP), Campinas (SP), Brazil.; II PT, PhD. Professor, Universidade Estadual de Campinas (UNICAMP), Campinas (SP), Brazil.; III MD, PhD. Pneumologist and Professor, Universidade Estadual de Campinas (UNICAMP), Campinas (SP), Brazil.; IV MD, PhD. Attending Physician and Assistant Lecturer, Universidade Estadual de Campinas (UNICAMP), Campinas (SP), Brazil.; V MD, PhD. General Surgeon and Professor, Universidade Estadual de Campinas (UNICAMP), Campinas (SP), Brazil.

**Keywords:** Obesity, Respiratory function tests, Carbon dioxide, Capnography, Leptin

## Abstract

**BACKGROUND:**

Excess trunk body fat in obese individuals influences respiratory physiological function. The aims of this study were to compare volumetric capnography findings (VCap) between severely obese patients and normal-weight subjects and to assess whether there is any association between neck circumference (NC), waist-hip ratio (WHR) and VCap among grade III obese individuals.

**DESIGN AND SETTING:**

Analytical observational case-matched cross-sectional study, University of Campinas.

**METHODS:**

This cross-sectional study compared VCap variables between 60 stage III obese patients and 60 normal-weight individuals.

**RESULTS:**

In comparison with the normal-weight group, obese patients presented higher alveolar minute volume (8.92 ± 4.94 versus 6.09 ± 2.2; P = < 0.0001), CO_2_ production (278 ± 91.0 versus 209 ± 60.23; P < 0.0001), expiratory tidal volume (807 ± 365 versus 624 ± 202; P = 0.005), CO_2_ production per breath (21.1 ± 9.7 versus 16.7 ± 6.16; P = 0.010) and peak expiratory flow (30.9 ± 11.9 versus 25.5 ± 9.13; P = 0.004). The end-expiratory CO_2_ (PetCO_2_) concentration (33.5 ± 4.88 versus 35.9 ± 3.79; P = 0.013) and the phase 3 slope were normalized according to expired tidal volume (0.02 ± 0.05 versus 0.03 ± 0.01; P = 0.049) were lower in the obese group.

**CONCLUSIONS:**

The greater the NC was, the larger were the alveolar minute volume, anatomical dead space, CO_2_ production per minute and per breath and expiratory volume; whereas the smaller were the phase 2 slope (P2Slp), phase 3 slope (P3Slp) and pressure drop in the mouth during inspiration.

## INTRODUCTION

Severely obese subjects develop various respiratory functional abnormalities, which may occur while awake, during sleep or while performing physical effort.^1^^,^^2^ There are obese individuals who hypoventilate during wakefulness, and this hypoventilation may cause hypoxemia and hypercapnia. These events characterize the diagnosis of alveolar hypoventilation in obese people. Not all severely obese individuals hypoventilate when awake, and the factors that favor this situation are not entirely clear.^3^^,^^4^^,^^5^ Among the respiratory abnormalities in obese patients that appear during sleep, the most common of these is obstructive sleep apnea caused by obstruction of the air inlet in the upper airway during inspiration.^4^^,^^5^

Obese subjects may also have difficulty performing physical effort because the accumulation of fat in the chest and abdomen may hinder the increased ventilation required by physical exertion.^4^ Besides alveolar hypoventilation, obese patients can show abnormalities of respiratory patterns even when they are not exercising, and little is known about such patterns.^4^^,^^5^

Pulse oximetry and measurement of arterial blood gases during wakefulness make it possible to diagnose situations of obesity hypoventilation syndrome.^4^^,^^5^ Polysomnography properly detects respiratory functional disorders that occur during sleep (obstructive apnea, central apnea and desaturation).^4^ However, spirometry requires physical effort and can show restrictive defects that are not necessarily related to involvement of the lungs. These restrictions may be caused by excessive accumulation of fat in the chest and abdomen that precludes proper spirometry maneuvers, which require physical effort.^6^^,^^7^^,^^8^^,^^9^

Volumetric capnography (VCap), which consists of plotting the expired CO_2_ concentration against expired volume, is a bedside-validated method for measuring airway and alveolar dead-space volumes, and ascertaining the pulmonary ventilation-perfusion relationships.^6^^,^^7^^,^^8^^,^^9^^,^^10^ In addition to its application in surgical and anesthetic settings, this method may be used among outpatients with chronic respiratory diseases.^11^^,^^12^ The examination is performed during spontaneous breathing, and breath-to-breath measurements are collected, including tidal volume, minute volume and end tidal CO_2_. Besides these variables, each breath cycle produces a curve called a capnogram that has a shape similar to that of N_2_ washout.

Our hypothesis was that VCap might help in understanding abnormalities in respiratory patterns and lung function during rest among obese individuals.^11^^,^^12^^,^^13^^,^^14^

The aims of this study were to compare the VCap variables between severely obese patients and normal-weight subjects and to evaluate the existence of any correlation between neck circumference (NC), waist/hip ratio (WHR) and VCap variables among obese patients.

## METHODS

This was an analytical observational case-matched cross-sectional study in which the subjects enrolled were grade III obese (body mass index, BMI ≥ 40 kg/m^2^) candidates for bariatric surgery at Hospital de Clínicas, University of Campinas (UNICAMP) and normal-weight controls (BMI 18.6-24.9) who were selected from the data bank of the Pulmonary Function Laboratory, UNICAMP. The subjects and controls were aged between 18 and 65 years old and were evaluated between August 2014 and July 2015. The study was approved by the local Research Ethics Committee.

The following potential participants were excluded: active smokers or former smokers who had quit less than one year previously; individuals with chronic respiratory diseases unrelated to obesity; vulnerable groups (individuals who were mentally ill, institutionalized or aged below 18 years); and individuals who opted not to take part in the study. Both the obese and the non-obese participants reported that they did not have any respiratory diseases (current or past) and were not on any respiratory medication.

The individuals selected were matched according to age, gender and height and were divided into two groups: obese (group 1) and non-obese (group 2). In both groups, the variables initially collected and analyzed were age, gender and anthropometric measurements (weight, height and BMI) (Table 1).

**Table 1:** Comparison of anthropometric variables between obese patients (group 1) and non-obese subjects (group 2), and data on frequencies of comorbidities, Berlin questionnaire (BQ) results, neck circumference and waist-hip ratio in group 1

	Obese (n = 60)		Non-obese (n = 60)	P-value
Women	45		45	1.0
Men	15		15	1.0
Age (years)*	36 ± 10		36 ± 11	0.92
Weight (kg)*	141 ± 27		67.3 ± 11	< 0.0001
Height (m)*	1.66 ± 0.09		1.66 ± 0.08	0.74
BMI (kg/m^2^)*	50.9 ± 7		20.8 ± 3	< 0.0001
NC*	44.1 (4.43)			
WHR*	0.91 (0.11)			
Arterial hypertension	27(55%)			
Diabetes	49 (81%)			
BQ results	High risk	Low risk		
	45	15		

BMI = body mass index; NC = neck circumference; WHR = waist-hip ratio. *Data presented as mean ± standard deviation. Statistical difference when P < 0.05.

In the obese group, the variables of NC and WHR were also collected and analyzed. The obese patients also answered the Berlin questionnaire (BQ), which evaluates the risk of development of obstructive sleep apnea syndrome (OSAS). Occurrences of hypertension were assessed in accordance with the Eighth National Joint Committee (JNC 8) criteria, i.e. individuals were considered to present hypertension if at least three different blood pressure measurements were higher than 140/90 mmHg.^15^ The presence of type 2 diabetes mellitus (T2DM) was assessed in accordance with the criteria defined by the current guidelines of the International Diabetes Federation (IDF). The presence of T2DM was defined as the presence of any of the following abnormalities: fasting plasma glucose ≥ 126 mg/dl; 75 g oral glucose tolerance test with fasting plasma glucose ≥ 126 mg/dl and/or two-hour plasma glucose ≥ 200 mg/dl; glycated hemoglobin (HbA1c) ≥ 6.5%; or random plasma glucose ≥ 200 mg/dl in the presence of classical diabetes symptoms.^16^

All the participants underwent VCap in the mornings at the Pulmonary Function Laboratory of UNICAMP. The device used was the oxy-capnograph and respiratory profile monitor (CO_2_SMOS Plus 8100 Dixtal/Novametrix; Respironics, Murrisville, PA, USA).

VCap is a technique that analyzes the pattern of CO_2_ elimination as a function of expired volume. It produces a curve called a capnogram, which represents the total amount of CO_2_ eliminated by the lungs during each breath. As would be expected, capnograms have the same shape as other gas elimination curves, with the advantage that they are obtained using a gas that is produced normally by the body and is eliminated by the lungs.^15^

The VCap examinations took about 10 minutes each and were always performed by the same technician. Firstly, the participant was asked to sit in a comfortable position and rest for five minutes while instructions for the examination were provided. Before the examination, the patient was encouraged to drink water to prevent the discomfort that could be caused by mouth breathing (e.g. dry throat, coughing, etc.). After this, a nose clip was put in place to avoid air escape through the nostrils and the subject was asked to breathe room air regularly through a mouthpiece that he or she held with his or her dominant hand, which was connected to the capnograph sensors. The participant was asked to breathe normally for some seconds to adapt to this situation.

Online digital recording of respiratory cycles was then started (Analysis Plus software), and this continued for five minutes. A pulse oximetry sensor was installed on the index finger. At the end of data collection, an offline sequence of the subjects’ respiratory cycles was selected to accommodate variation of < 15% for expiratory tidal volume and < 5% for partial pressure of CO_2_ concentration (PetCO_2_). Furthermore, sequences that showed phases 2 and 3 slopes equal to zero were eliminated, along with outlier values.

Phases 2 and 3 slopes equal to zero can occur because, from time to time, the apparatus used removes the humidity from the sensor and, through this, the parameters are cleared. Outlier values occur because patients who are breathing spontaneously may make different respiratory efforts (e.g. sighing, coughing or speaking, etc.).

The patients were weighed using a digital scale that was appropriate for their size, with a maximum capacity of 300 kg and a resolution of 100 g, and height was ascertained using a wall-mounted stadiometer. From these measurements, BMI in kg/m^2^ was calculated. 

Waist circumference was determined using a measuring tape around the individual at the natural waist line, i.e. in the narrowest area between the chest and hip, at the midpoint between the last rib and the iliac crest. The readings were made at the end of expiration. Hip circumference was determined in the horizontal plane at the level of the greatest posterior protuberance of the buttocks. The waist/hip ratio (WHR) was calculated using the formula: WHR = waist circumference (cm)/hip circumference (cm). Both of these measurements were made with the patient standing, using a flexible measuring tape. Neck circumference was also measured using a flexible tape at the level of the laryngeal prominence.

### Statistical analysis

The baseline characteristics of the sample were presented in frequency tables encompassing the categorical variables, as absolute (n) and percentage (%) frequencies. Continuous variables were presented as mean values with their respective standard deviations (SD). Bivariate analyses on categorical variables were carried out through the chi-square and Fisher’s exact tests. For comparisons of continuous measurements, the Mann-Whitney test was used. To study the relationship between the capnography measurements and, respectively, the neck circumference and the waist-to-hip ratio of the obese individuals, Spearman’s correlation coefficient was used. The significance level used was 5% (P < 0.05). A Bonferroni adjustment was used for pairwise comparisons, to identify the significance within mean differences between correlations for each outcome (Bonferroni adjusted α = 0.0015625 for Mann-Whitney comparisons and α = 0.0045 for Spearman’s correlations). To perform the analyses, the Statistical Analysis System (SAS) software for Windows, version 9.2, was used.

Sample size estimation was performed through a single-proportion formula with a 95% confidence interval. Precision was set at 10% and the calculated sample size was determined to be 60.

## RESULTS

In the obese group, there were 45 women (75%) and 15 men (25%). Their mean age was 36 years, mean weight 141 kg, mean height 1.66 m, mean BMI 50.9 kg/m^2^, NC 44 cm and WHR 0.91. These two last anthropometric parameters were only measured in the group of obese individuals.

In this group, 27 (55%) had hypertension and 49 (81.7%) were diabetic. In the Berlin Questionnaire, 45 individuals (75%) presented a high risk of having OSAS and 15 (25%) presented a low risk.

In the non-obese group, 45 (75%) were women and 15 (25%) were men, with a mean age of 36 years, mean weight 67.3 kg, mean height 1.66 m and mean BMI 20.8 kg/m^2^. Table 1 summarizes the baseline characteristics of all the patients.

The obese patients presented alveolar minute volume (MV alv) (ml), production of CO_2_ per minute (VCO_2_) (ml/min), partial pressure of end-tidal carbon dioxide (PetCO_2_) (mmHg), expiratory volume (Ve) (ml), peak expiratory flow (PEF) (l/min), production of CO_2_ per breath (VCO_2_/br) (ml/breath) and phase 3 slope normalized according to end-tidal partial pressure of CO_2_ (P3Slp/Ve) that were statistically different (P < 0.05) from those of the normal-weight controls. After applying the Holm-Bonferroni correction, MV alv (ml) and VCO_2_ (ml/min) remained significantly higher in the obese group (P < 0.0015625). The capnography variables are shown in Table 2.

**Table 2:** Distribution of the variables of age, anthropometry and volumetric capnography (VCap) among patients with and without obesity

Variables	Obese			Non-obese			P-value
	Average	SD	Median	Average	SD	Median	
RR (cpm)	14	4.94	14.1	13	3.76	13.4	0.365
HR (bpm)	74	13.7	74.7	77	11.3	76.5	0.198
SpO_2_ (%)	97	1.02	97.4	97	0.64	97.8	0.155
VD (aw) (ml)	134	35.5	134	137	28.4	131	0.639
PeCO_2_ (mmHg)	24.2	3.56	24.3	24	3.49	24.1	0.661
Vi (ml)	760	349	717	622	203	590	0.053
Ti (sec)	1.91	0.66	1.66	1.87	0.56	1.72	0.950
Te (sec)	2.66	0.98	2.46	2.87	0.97	2.81	0.129
PIF (l/min)	35.1	13.3	32.8	31.8	8.7	31.3	0.307
RSBI (VT/RR)	26.4	21.9	21.2	25.7	14.2	23.4	0.430
P2Slp (mmHg/l)	333	156	297	337	82.5	331	0.224
P3Slp (mmHg/l)	10.2	9.19	7.32	10.9	15.0	8.16	0.286
P3Slp/PetCO_2_ (mmHg)	0.3	0.24	0.24	0.31	0.48	0.23	0.803
NIP (cmH_2_O)	-0.36	0.65	-0.05	-0.7	4.27	-0.02	0.243
MV alv (ml)	8.92	4.15	8	6.09	2.2	5.3	< 0.0001
VCO_2_ (ml/min)	278	91.0	268	209	60.2	199	< 0.0001
PetCO_2_ (mmHg)	33.5	4.88	33.9	35.9	3.79	35.8	0.013
Ve (ml)	807	365	763	624	202	587	0.005
PEF (l/min)	30.9	11.9	28.6	25.5	9.13	23.3	0.004
VCO_2_/br (ml/breath)	21.1	9.7	19.3	16.7	6.16	15.7	0.010
P3Slp/Ve	0.02	0.05	0.01	0.03	0.09	0.01	0.049

RR = respiratory rate; HR = heart rate; SpO_2_ = oxygen saturation; VD (aw) = anatomical dead space; PeCO_2_ = mean end-tidal partial pressure of CO_2_; Vi = inspiratory tidal volume; Ti = inspiratory time; Te = expiratory time; PIF = peak inspiratory flow; RSBI = Tobin index; VT = tidal volume; P2Slp = phase 2 slope; P3Slp = phase 3 slope; P3Slp/PetCO_2 =_ phase 3 slope normalized according to end-tidal partial pressure of CO_2_; NIP = negative inspiratory pressure; MV alv = alveolar minute volume; VCO_2_ = CO_2_ production; PetCO_2_ = end-tidal partial pressure of CO_2_; Ve = expiratory tidal volume; PEF = peak expiratory flow; VCO_2_/br = CO_2_ production per breath; P3Slp/Ve = phase 3 slope normalized according to expired tidal volume. Results are presented as means, medians and standard deviations. P-value for the Mann-Whitney test comparing group 1 (obese) with group 2 (non-obese) after the Bonferroni adjustment; P < 0.0015625.

In the obese individuals, WHR did not present any correlation with VCap measurements. On the other hand, in correlation analyses between NC measurements and capnography variables (Table 3), there were significant correlations for the following variables: the greater the NC was, the larger were the alveolar minute volume (MV alv), the anatomical dead space (V_D_), the production of CO_2_ per minute (VCO_2_), the inspiratory volume (Vi), the expiratory volume (Ve), the peak expiratory flow (PEF) and the production of CO_2_ per breath (VCO_2_/br); whereas the greater the NC was, the smaller were the phase 2 slope (P2Slp), the phase 3 slope (P3Slp) and the pressure drop in the mouth during negative inspiratory pressure (NIP). After applying the Bonferroni adjustment (P < 0.0045), NIP, P3Slp and VCO_2_/br no longer presented significant correlations, while the other variables remained significant.

**Table 3:** Analysis on correlations between neck circumference (NC) and volumetric capnography (VCap) variables among obese individuals (Group 1)

VCap variables	r	P
MV alv (l)	0.43	0.0006
VD (aw) (ml)	0.45	0.0003
VCO_2_ (ml/min)	0.48	< 0.0001
Vi (ml)	0.36	0.0045
Ve (ml)	0.36	0.0041
PEF (l/min)	0.40	0.0017
VCO_2_/br (ml/breath)	0.34	0.0082
P2Slp (mmHg/l)	-0.38	0.0027
P3Slp /Ve	-0.29	0.0242
PIF (l/min)	0.47	< 0.0001
NIP (cmH_2_O)	-0.33	0.0100

MV alv (L) = alveolar minute volume; VD (aw) (ml) = anatomical dead space; VCO_2_ (ml/min) = CO_2_ production; Vi (mL) = inspiratory tidal volume; Ve (ml) = expiratory tidal volume; PEF (l/min) = peak expiratory flow; VCO_2_/br (ml/breath) = CO_2_ production per breath; P2Slp (mmHg/l) = phase 2 slope; P3Slp (mmHg/l) = phase 3 slope; PIF (l/min) = peak inspiratory flow; NIP (cmH_2_O) = negative inspiratory pressure. P-value results regarding the Spearman correlation test are from correlations between NC and WHR measurements and the VCap variables of the obese individuals, adjusted in accordance with the Bonferroni correction: P < 0.0045.

## DISCUSSION

The obese patients in this study had significantly greater alveolar minute volume (MV alv) and expired volume of CO_2_ (average per minute) or VCO_2_. These results (greater MV alv with equal respiratory rate) suggest that obese patients possibly have more efficient ventilation during tidal breathing, since the VCap measurements are made during several minutes of quiet breathing. These measurements do not require forced expiration, as spirometry does. Tidal volume and alveolar minute ventilation may be higher in order to match the higher needs of a greater body mass. Nevertheless, the low mean PetCO_2_ and P3Slp/Ve may indicate that the obese group presented a larger alveolated area. The comparison between the two groups (obese versus non-obese subjects) revealed that there were no statistical differences regarding age and height. The respiratory rate and the dead volume of the airways were similar in the two groups.

The reductions in forced vital capacity that are detected in obese patients through spirometry may, at least in part, be related to the difficulties in using expiratory abdominal muscles due to fat accumulation in this area. The obese patients in the present study did not perform spirometry because this was not part of the study protocol.

Wei et al. found that the lungs of obese patients had higher capacity to diffuse carbon monoxide and attributed this result to the higher blood flow to the lungs that obese subjects may have.^14^ However, our results may suggest that their greater capacity for diffusion may be due to a real increase in alveolar surface area.

The peripheral airspaces have been termed the silent lung zone because conventional lung function tests are unable to detect their involvement in disease processes. Inert gases such as helium (He), nitrogen (N_2_) and sulfur hexafluoride (SF_6_) have been used to study these areas and the elimination curves that they produce during each expiration depict concentrations of the gas at different volumes until all the expiratory volume has been expired. Irrespective of the gas used, all curves have the same shape and, on each of them, three phases can be identified: phase 1, with very low concentrations of the gas, corresponds to the elimination of the air from the anatomical dead space; phase 2, which is generally a steep upward line, represents the growing concentration of the gas that is eliminated from proximal alveolated air spaces; and phase 3, which is almost a plateau line, represents the elimination of the gas from most of the alveoli in the lungs. The phase 3 slope (mmHg/l) is an important feature of gas washout curves and contains information about gas transportation in the alveolated airways of the lung periphery. It varies in many pathological conditions of the lungs.^14^^,^^15^^,^^16^^,^^17^^,^^18^^,^^19^^,^^20^^,^^21^

Phase 3 (mmHg/l) of the VCap represents the elimination of CO_2_ from most of the alveoli and, in normal individuals, it is almost a plateau, with a slight upward slope. The phase 3 slope (P3Slp) should therefore be small.

Because of the very small convective velocities in the lung periphery, gas transportation through diffusion is the dominant mechanism in acinar air spaces. Steepened phase 3 slopes may represent increased diffusional resistance in the peripheral lung. These steepened slopes occur when breathing involves a smaller-than-normal maximum interfacial area between the tidal volume and the functional residual capacity (FRC) during a breathing interval. Small tidal volumes produce steeper slopes because the inhaled air penetrates to shallower-than-normal depths in the lung and therefore encounters smaller-than-normal maximum interfacial areas and longer gas-phase diffusion paths.^22^^,^^23^^,^^24^ Hence, there is a need to normalize phase 3 slopes when comparing subjects with significantly different expired volumes.^11^^,^^12^

In a paper on VCap in children, Ream et al.^25^ postulated that the observed decrease in normalized phase 3 slopes of CO_2_ washout curves with increasing age was due to an increase in the interface between functional residual capacity (FRC) and tidal volume (VT), with lung growth in children.

It seems that, taking into account the significantly smaller normalized values of phase 3 slopes in obese patients, a hypothesis can be put forward: obese patients may have larger or more efficient alveolated airspaces than do non-obese subjects. The correlations between NC values and capnography variables that were found to be significant are in agreement with the hypothesis put forward here, i.e. that the greater the body mass was (reflected in this analysis by greater NC), the larger were the lungs of the obese subjects who participated in this study.

Human leptin is a 16-kDa protein of 167 amino acids and was the first fat cell-derived hormone to be discovered.^26^^,^^27^ Leptin is produced primarily in the adipocytes of white adipose tissue. In the fetal lungs, leptin is induced in a special phenotype of alveolar interstitial fibroblasts, called lipofibroblasts, through the action of the parathyroid hormone-related protein (PTHrP), which is secreted by the alveolar epithelium under moderate stretching. The leptin from the mesenchyme, in turn, acts back on the epithelium at the leptin receptor carried in the alveolar type II pneumocytes and induces surfactant expression, which is of paramount importance for lung function.^28^^,^^29^^,^^30^ Leptin-deficient mice show altered postnatal lung development. They have reduced lung volume and alveolar surface area and the alveolar size does not increase with age.^31^

Leptin levels are paradoxically increased in situations of obesity.^32^ Although, as a circulating signal, leptin reduces appetite, obese individuals generally exhibit higher circulating concentrations of leptin than do normal-weight individuals, because of their higher percentage of body fat. The expected responses to high levels of leptin are decreased calorie intake and increased energy expenditure. Most obese humans are probably insensitive to this action by leptin, and behave similarly to type 2 diabetic patients who show resistance to the action of insulin.

The excess leptin present in obese subjects may at least partly explain the apparently larger lungs detected through VCap in these patients. Fat deposits in the neck and abdomen may display a mechanical effect on the chest and may hinder chest mobility during forced expiration and possibly also during spontaneous ventilation, with consequences for lung function. This has also been observed in other studies,^33^^,^^34^^,^^35^^,^^36^^,^^37^ such as the increased risk of developing lung-base atelectasis in obese individuals due to mechanical compression exerted by the abdomen in the region.^38^ Greater NC can cause increased airflow resistance, especially when NC is greater than 40 cm, and this can result in hypoventilation.

None of the obese patients in the present study, whose BMI was greater than 52, had obesity hypoventilation syndrome (OHS), although 45 of them showed a high-risk result from the Berlin questionnaire for obstructive sleep apnea syndrome (OSAS).^39^ The scores in the Berlin questionnaire were positively correlated with neck circumference. No sleep studies were available for these individuals.

The hypothesis that these individuals may have had a larger alveolar-capillary membrane might serve as a possible reason for the absence of CO_2_ retention. Recent reports have shown that alveolar growth continues from childhood into adolescence in humans and other mammals.^33-35^ The lungs of obese patients who were obese children may have been exposed to higher levels of leptin during a period when the alveoli were actively growing. Obesity hypoventilation syndrome might perhaps be more commonly found among obese subjects whose weight gain occurred later in life, a hypothesis that awaits further investigation.

Ferreira et al.^40^ analyzed obese and normal-weight children without asthma and reported that the obese children had greater lung volumes, shown through volumetric capnography, and lower values for the ratio between the phase 3 slope of the volumetric capnogram and the expired volume (P3Slp/Ve). These findings were consistent with the results obtained by Ream et al.^25^ who evaluated infants, children and adolescents and found lower values for P3Slp in individuals with higher Ve, and a negative association between P3Slp, body weight and body surface area. Growth increased the number of alveoli in the lungs and reduced the phase 3 slope on capnograms. The obese children in the study by Ferreira et al. seemed to have larger lungs.

### Limitations and strength

This study has some limitations. Carbon monoxide diffusion was not performed on the patients of the present study because we did not have the equipment to do this. This measurement could have helped to rule out alterations in carbon monoxide diffusion as the cause of our findings. The NC and WHR data relating to the controls were not available because the data were collected from a database. Spirometry data were not available because the obese patients were in the process of losing weight and would undergo spirometry after achieving a predetermined goal. It was not possible to evaluate whether exposure to toxic agents such as tobacco might have influenced respiratory function in the two groups, since we did not have this information in the database relating to the non-obese group.

Our findings may contribute towards understanding the complex range of alterations to respiratory function that are seen in very obese patients. They also raise the intriguing possibility that some obese individuals may undergo changes to the alveolar-capillary membrane that enable greater efficiency of CO_2_ elimination. Knowing the leptin levels in these patients might have contributed towards investigating this hypothesis.

## CONCLUSION

Volumetric capnography was capable of detecting particular characteristics of lung structure and function in morbidly obese patients, among which some had not previously been shown. It identified changes to various respiratory parameters, in comparison with a normal-weight control group. The greater the NC was, the larger were the alveolar minute volume, anatomical dead space, CO_2_ production per minute and per breath and expiratory volume; whereas the smaller were the phase 2 slope (P2Slp), phase 3 slope (P3Slp) and pressure drop in the mouth during inspiration. Some of these changes were related to possibly larger lungs in obese patients and others to their larger NC. The combination of larger lungs and absence of hypoventilation makes us speculate that these two occurrences may be linked.
